# Patterns of Understory Diversity in Mixed Coniferous Forests of Southern California Impacted by Air Pollution

**DOI:** 10.1100/tsw.2007.72

**Published:** 2007-03-21

**Authors:** Edith B. Allen, Patrick J. Temple, Andrzej Bytnerowicz, Michael J. Arbaugh, Abby G. Sirulnik, Leela E. Rao

**Affiliations:** ^1^ Department of Botany and Plant Sciences and Center for Conservation Biology, University of California, Riverside, CA, USA; ^2^ USDA Forest Service, Pacific Southwest Research Station, Riverside, CA, USA; ^3^ Department of Environmental Sciences, University of California, Riverside, CA, USA

**Keywords:** nitrogen deposition and loss of plant species richness, ozone impacts on herbaceous plants, long-term changes in plant species richness, southern California mixed coniferous forest

## Abstract

The forests of the San Bernardino Mountains have been subject to ozone and nitrogen (N) deposition for some 60 years. Much work has been done to assess the impacts of these pollutants on trees, but little is known about how the diverse understory flora has fared. Understory vegetation has declined in diversity in response to elevated N in the eastern U.S. and Europe. Six sites along an ozone and N deposition gradient that had been part of a long-term study on response of plants to air pollution beginning in 1973 were resampled in 2003. Historic ozone data and leaf injury scores confirmed the gradient. Present-day ozone levels were almost half of these, and recent atmospheric N pollution concentrations confirmed the continued air pollution gradient. Both total and extractable soil N were higher in sites on the western end of the gradient closer to the urban source of pollution, pH was lower, and soil carbon (C) and litter were higher. The gradient also had decreasing precipitation and increasing elevation from west to east. However, the dominant tree species were the same across the gradient.

Tree basal area increased during the 30-year interval in five of the sites. The two westernmost sites had 30–45% cover divided equally between native and exotic understory herbaceous species, while the other sites had only 3–13% cover dominated by native species. The high production is likely related to higher precipitation at the western sites as well as elevated N. The species richness was in the range of 24 to 30 in four of the sites, but one site of intermediate N deposition had 42 species, while the easternmost, least polluted site had 57 species. These were primarily native species, as no site had more than one to three exotic species. In three of six sites, 20–40% of species were lost between 1973 and 2003, including the two westernmost sites. Two sites with intermediate pollution had little change in total species number over 30 years, and the easternmost site had more species in 2003. The easternmost site is also the driest and has the most sunlight filtering to the forest floor, possibly accounting for the higher species richness. The confounding effects of the precipitation gradient and possibly local disturbances do not show a simple correlation of air pollution with patterns of native and invasive species cover and richness. Nevertheless, the decline of native species and dominance by exotic species in the two westernmost polluted sites is cause for concern that air pollution is affecting the understory vegetation adversely.

### INTRODUCTION

There is considerable evidence from the eastern U.S. and Europe that nitrogenous (N) and ozone air pollution are impacting natural vegetation survival and diversity[1,2,3]. The impacts of ozone were recognized for tree mortality in southern California coniferous forests, but potentially negative impacts of anthropogenic N deposition have been less evident in these forests. Historically, high concentrations of ozone caused plant mortality, as occurred for trees in the coniferous forests surrounding the Los Angeles air basin during the 1960s and 1970s[4,5]. Since then, air pollution regulations have reduced the concentrations of ozone so that acute levels leading to rapid mortality are not seen in the U.S.[5]. However, symptoms of ozone damage are still observed, indicating that chronic levels that may cause long-term vegetation changes still occur[3].

In contrast to ozone, the impacts of anthropogenic N deposition on vegetation has increased globally and locally[2,6]. Nitrogen deposition to shrublands and forests of the Los Angeles air basin may be as high as 30–50 kg $ha^{-1}$ $year^{-1}$[7,8,9,10]. Most of this arrives as dry deposition in gaseous, ionic, and particulate forms of oxidized and reduced N during the dry summer season. Plant-available forms of N accumulate on plant and soil surfaces until the following rainy season, when they are leached to the rooting zone for plant uptake[11]. The major effect of N deposition on trees has been to increase their growth rate[12], which may be considered beneficial in terms of forest production. However, high levels of ozone co-occur with high concentrations of oxidized N, so multiple impacts occur simultaneously. Ozone causes needle drop in pines that increases the litter depth, while elevated N may act as a fertilizer and continue to promote growth of trees negatively affected by ozone[13,14].

Few studies have examined the impacts of air pollution on forest understory species[2,3]. Ozone impacts on herbs and shrubs have been of lesser interest because they form a relatively small proportion of forest productivity, and have historically not been included in most studies. Ozone damage is more difficult to quantify on a community basis than for trees because of higher population turnover rates of herbs of the different species. Alternatively, herbs have thinner leaves and higher rates of photosynthesis than most trees, and may be more sensitive to ozone[3]. The understory herbs tend to be high in diversity in mixed coniferous ecosystems, compared to the relatively few tree species in any stand. An assessment of understory impacts from air pollution is important from a standpoint of biodiversity conservation, both for the sake of plant diversity and for the many animal species that depend on them. Chronic impacts of ozone are still occurring in the San Bernardino Mountains where several species of shrubs and forbs, but no grasses, were observed with ozone-injury symptoms during a 1996 survey[3]. These impacts occurred in the western San Bernardino Mountains where the ozone concentrations are highest.

The impacts of elevated N have also not been quantified for the forest understory productivity or diversity in the San Bernardino Mountains. Studies in eastern U.S. and European deciduous forests have shown a mixed response of understory diversity to N deposition or fertilization[2], with no significant response in sites naturally high in soil N or already receiving anthropogenic N deposition. Other studies from shrub, forb, and grasslands from various regions have shown the negative effects of N deposition on herbaceous plant diversity. In the Netherlands and the U.K., native herbaceous species lost diversity while native grasses increased in biomass with N deposition[15,16]. Nitrogen fertilizer studies in the Mojave Desert[17,18] and coastal sage scrub[19] have shown increased productivity of invasive grasses, and decreased productivity and diversity of native species. The diversity of native forb species along a N

deposition gradient in coastal sage scrub decreased from 70 per hectare at sites with low air pollution, to 20 per hectare at polluted sites[20, and unpublished observations]. A major difference between the continents is that the European studies documented native grass production[15], while in the U.S., where many European and Asian grasses have invaded[21], the loss of native diversity comes at the expense of increases in the invasive species[17,18,19,20].

For the last 60 years, the forests of the San Bernardino Mountains of southern California have been exposed to some of the highest rates of N and ozone deposition of any forest ecosystem in the world. A large multidisciplinary task force was assembled in 1973 to study the impacts of ozone on the mixed conifer forest, and a series of 18 permanent pine plots was established running west to east along an ozone and N pollution gradient. Measurements of ozone, N deposition, other environmental variables, and tree responses along the gradient from 1974 until 1998 were given in a series of publications[5]. The 1973 survey included the understory species that were present in these plots[22], but no other data to assess air pollution impacts on herbaceous species have been collected, with the exception of Temple et al.'s survey of ozone symptoms in 1996[3]. In 2003, we assessed tree and understory vegetation in a subset of six of the historic 1973 plots to determine changes in the understory vegetation by species. Several invasive species have become abundant in the San Bernardino Mountains that were noted both in the historic and recent surveys. To determine the characteristics of the air pollution gradient, historic and present-day air pollution data were assembled, and soils were analyzed for N, C, and pH. The present-day understory species composition was compared with the historic species list, and is discussed in relation to changes in the atmosphere and soil along the gradient.

### METHODS

Six sites were chosen along a west-to-east air pollution gradient in the San Bernardino National Forest (SBNF) that has been under investigation since 1973 (Fig. 1). Originally there were 18 sites, but these varied greatly in vegetation, elevation, and precipitation[22], so this subset of six was chosen to be as similar in forest species composition as possible, and to minimize the elevation and precipitation gradient. Nevertheless, there is a moisture gradient from west to east in the San Bernardino Mountains that is reflected in the six sites, which also increases in elevation from west to east (Table 1). The westernmost site, Barton Flats, is driest while precipitation is higher at all the other sites. Precipitation data have not been collected at all of the sites since intensive investigation from 1973–1977, and precipitation for this time period was uncharacteristically low for Camp Paivika. Long-term precipitation data indicate Camp Paivika is a mesic site, while the recent data for Barton Flats continue to reflect that it is relatively dry (Table 1[23]). Recent data are not available for other sites. The sites have old-growth forest and have been protected as research sites. All sites are >200 m distant from settlements and roads. Some may have received incidental foot traffic, but there was no perceptible damage to understory vegetation during the 2003 field season.

The air pollution gradient is indicated by historic and current ozone data, and present-day atmospheric reactive N concentrations. Ozone data from 1974–1978 show a gradient from west to east, with 24-h averages of 110 ppb at Camp Paivika and 70 ppb at Barton Flats[24]. Hourly concentrations at Camp Paivika were up to 600 ppb during mid-day in those years[3]. The degree of ozone injury to pine needles reflected the ozone concentration gradient (greatest injury at Camp Paivika and lowest at Barton Flats and Camp Angeles[24]). Ozone concentrations today are over 50% lower than 30 years ago, but ozone injury can still be observed, especially for pines at Camp Paivika and Breezy Point[3]. Measurements of N components of air pollution were measured at these sites recently. Measured atmospheric concentrations of nitric acid in 2002–2005 ranged from 6.6 $\mu g\ m^{-3}$ at Camp Paivika to 2.1 $\mu g\ m^{-3}$ at Barton Flats, and $NH_3$ was lower, but with the same pattern (Table 2[25]). Dogwood has levels of ozone and N pollution similar to Camp Paivika, both historically and currently. Nitrogen deposition, including wet plus dry deposition, has been measured at the two extreme sites, and ranges from 25–35 kg N $ha^{-1}\ year^{-1}$ at Camp Paivika to 3–6 kg $ha^{-1}\ year^{-1}$ at Barton Flats[23].

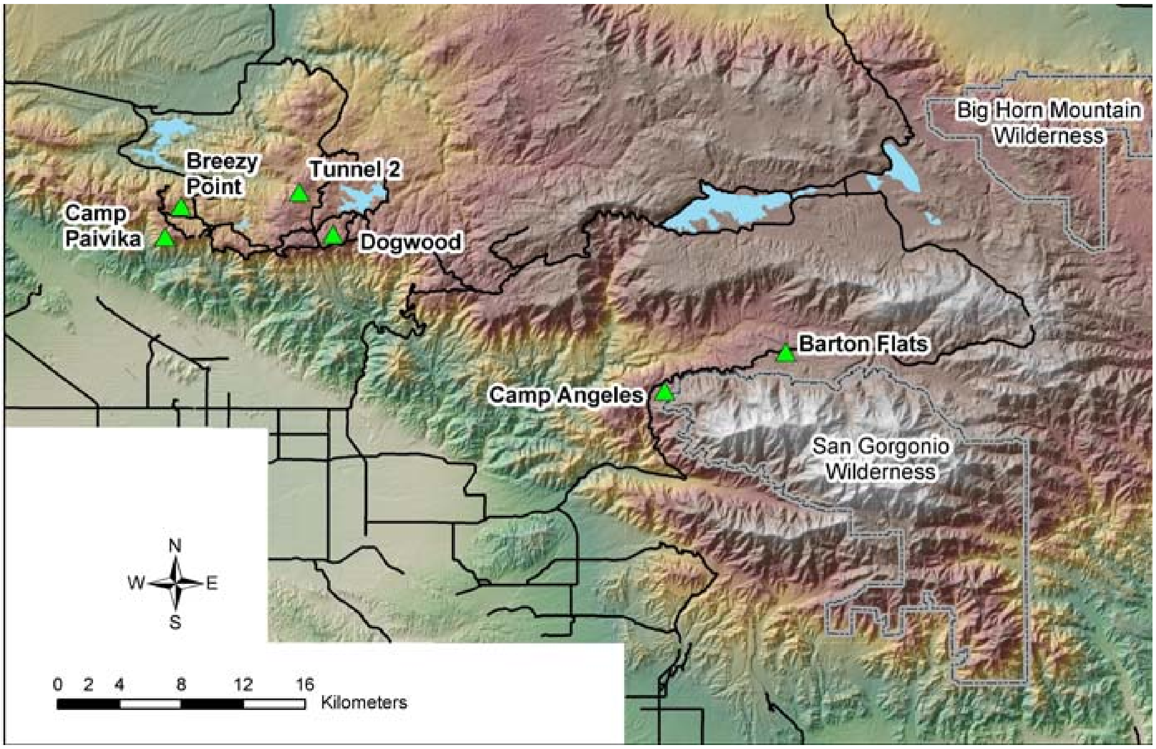

**FIGURE 1.** Locations of six sample sites along an air pollution gradient in the San Bernardino Mountains, with greatest pollution occurring in the western sites. Prevailing winds are from the west.

**TABLE 1**
**Characteristics of Sample Sites in the San Bernardino Mountains**

| Plot Name | Elevation (m) | Precip (cm)* | Lat | Long |
|---|---|---|---|---|
| Camp Paivika | 1600 | 78 (98)** | 34° 14’ 05” | 117° 19’ 25” |
| Breezy Point | 1525 | 105 | 34° 15’ 20” | 117° 18’ 30” |
| Dogwood | 1725 | 90 | 34° 14’ 15” | 117° 13’ 00” |
| Tunnel Two | 1640 | 90 | 34° 16’ 10” | 117° 13’ 15” |
| Camp Angeles | 1830 | 114 | 34° 08’ 30” | 116° 58’ 40” |
| Barton Flats | 1900 | 61 (61)** | 34° 10’ 10” | 116 ° 54’ 25” |

*Note:* Arranged from high to low pollution (W to E). Mean annual temperature ranges from 12.9°C at Camp Paivika to 10.6° at Barton Flats.

* Precipitation values from 1973–1977[24].

** Values in parentheses from 1956–1991 for Camp Paivika, 1992–1997 for Barton Flats[23].

Vegetation data collected during the 1970s emphasized the canopy, but included a list of understory species. The six sites were dominated by mixed coniferous forest with ponderosa pine, Jeffrey pine, white fir, and Kellogg oak. The major objectives of the study were to determine the impacts of air pollutants on tree growth and physiology as well as changes to soils [summarized in 5]. At each site, 50 pines of >10 cm diameter at breast height (dbh) were marked along three transects. The length of each transect was variable,

**TABLE 2**
**Historic Ozone Atmospheric Concentrations in SBNF Pine Plots[24] and Recent Ozone, Nitric Acid Vapor, and Ammonia[25]**

| Site | Ozone* 1974–1978[a] ppb | Injury Score[b] | Ozone* 2002–2005[c] ppb (S.E.) | HNO3 2002–2005[c] µg $m^{-3}$ (S.E.) | NH3 2002–2005[c] µg $m^{-3}$ (S.E.) |
|---|---|---|---|---|---|
| Camp Paivika | 110 | 13 | 63.2 (5.3) | 6.6 (1.1) | 5.4 (1.4) |
| Breezy Point | 90 | 15.2 | 56.6 (6.2) | 4.6 (1.3) | 4.1 (1.0) |
| Dogwood[d] | 100 | 19.8 | 63.3 (8.4) | 7.2 (0.9) | 5.4 (0.9) |
| Tunnel Two[e] | 100 | 21.9 | 55.8 (7.4) | 3.5 (1.4) | 3.7 (0.7) |
| Camp Angeles | 90 | 22.5 | 56.9 (6.3) | 3.0 (0.8) | 4.1 (0.7) |
| Barton Flats | 70 | 26.1 | 52.4 (4.8) | 2.1 (0.5) | 3.6 (0.5) |

* 2002–2005 data collected using passive samplers have been calibrated against UV absorption active monitors used by Miller et al. from 1974–1978[24].

[a] Average hourly ozone concentration over 24 h, May–October, 1974–1978.

[b] Low injury score = high ozone injury.

[c] Mean concentrations of air pollutants measured with passive samplers. Collection periods: May 20 to October 20, 2002; June 12 to November 20, 2003; June 3 to November 5, 2004; May 4 to October 4, 2005.

[d] For the 2002–2005 samples, data were collected at Rim of the World, 4 km south of Dogwood. For Breezy Point in 2004, concentrations of pollutants are estimated based on comparisons with Camp Paivika and Rim of the World in 2002, 2003, and 2005.

[e] For Tunnel Two in 2002, concentrations of pollutants are estimated based on comparisons with the neighboring site of Rim of the World.

depending on the density and size of trees at each site, resulting in variable plot sizes. The plot sizes were not recorded, but based on a hand-drawn map from Breezy Point[22] were on the order of 100 m on one side, possibly less than 1 ha. Understory vegetation was sampled in each pine plot by listing all the plant species found in a linear transect along the length of each plot. Tree and shrub data included basal area, density, percent cover, and frequency, but herbaceous species were only recorded as present, but not otherwise quantified[26].

Understory vegetation cover was assessed in three 1-ha plots at each of the six sites during 2 weeks in June 2003. Percent cover was estimated in 50, 0.25-$m^2$ quadrats in each hectare, or a total of 150 quadrats per site. The quadrats were located at 5-m intervals along five, 50-m transects in each hectare. Cover estimations were made to 1% in gridded 0.5- × 0.5-m frames, although tiny forbs were estimated to 0.1%. In addition, a list was made of all species present in the 3 ha by walking through the entire area. Percent cover of native and exotic species, and native species richness, were compared statistically among the six sites. In addition, the species lists for 1973 were compared with the lists for 2003 for each site. Shrub species were not included in the analysis as shrub cover was low in the plots (<1%) and may have been undersampled using 0.25-$m^2$ quadrats, although subshrubs were included.

Tree density and basal area were also assessed in five, 5- × 50-m belt transects in each of the 1-ha plots, or a total of 15 belt transects per site. The diameter was measured for each tree >10 cm dbh and basal area calculated. Trees <10 cm dbh were counted by species. These data were compared with data from 1973.

Soil samples were collected in August 2006 to determine soil N, C, pH, and litter depth. Tree mortality following the 2002 record drought year occurred to some extent in most of the sites. Fire in October 2003 burned part of the Camp Paivika site, and dead tree and brush clearing were done to reduce the fire hazard at

Camp Paivika, Tunnel Two, and Camp Angeles. In some cases, the exact location of the 2003 vegetation transects could not be resampled, so samples were collected from undisturbed forest sites as close as possible to the original vegetation sampling locations in undisturbed soils. Litter was removed from 30- × 30-cm quadrats at nine locations at each site and litter depth was recorded. Three soil cores of 2.5-cm diameter were taken from each quadrat in the mineral soil to 5-cm depth and bulked, for a total of nine bulked samples per site. The soils were measured for pH (50:50 soil:distilled water paste), total C and N by combustion with a CNS Analyzer, and KCl-extractable ammonium and nitrate with a Technicon Autoanalzyer. Chemical analyses were done by the University of California, Davis, Analytical Laboratory.

### RESULTS

#### Soils

Analyses of soil N, C, pH, and litter from 2006 samples showed a gradient from high to low values among the sites lying from west to east along the air pollution gradient. Total extractable inorganic N was significantly higher at the westernmost site, Camp Paivika, than the five other sites (Fig. 2A). When ammonium was analyzed separately, it was significantly higher at Camp Paivika than all the other sites except the adjacent Breezy Point ($p = 0.016$), while nitrate was significantly higher at Camp Paivika than all the other sites ($p = 0.0001$). pH was lowest at Camp Paivika and highest at the three easternmost sites (Fig. 2B). Total N was highest at Camp Paivika, intermediate at Breezy Point, Dogwood, and Tunnel Two, and significantly higher at Camp Paivika than the two eastern-most sites, Camp Angeles and Barton Flats (Fig. 3A). Total soil C followed a similar pattern, with significantly lower values for Camp Angeles and Barton Flats than Camp Paivika (Fig. 3B). Litter depth was greatest at Camp Paivika, intermediate at Breezy Point, and significantly lower at the other four sites (Fig. 4).

#### Vegetation

Density of trees > 10 cm dbh increased in all of the sites between 1973 and 2003 (Table 3). Tree basal area (calculated from trees >10 cm dbh) increased in five of the six sites between 1973 and 2003; the slight decline at Camp Angeles is likely due to sampling error (Table 3). Dogwood had the greatest density of trees of size classes < and >10 dbh in 2003. *Pinus ponderosa* was the most abundant tree at all of the sites in both years, and *Quercus kelloggi* was the second most abundant (data not shown). *Pinus jeffreyi* was codominant with *P. ponderosa* at Barton Flats, but was absent at other sites. *Calocedrus decurrens* occurred at all the sites in 2003 with varying dominance, *Abies concolor* occurred at all sites but Camp Paivika, *P. lambertiana* occurred at all sites but Breezy Point, *P. coulteri* occurred only at Tunnel Two, and *Q. chrysolepis* occurred only at Breezy Point.

The percent cover of both native and exotic native herbaceous species in 2003 was greatest at the two westernmost sites, possibly reflecting the higher productivity of the sites (Fig. 5A). Breezy Point and Camp Paivika had an especially high cover of exotic species due primarily to annual *Galium aparine*, with a small amount of *Bromus diandrus*. Dogwood, Tunnel Two, and Camp Angeles all had less than 5% total cover with very little contribution from exotic species. Barton Flats had 11% cover of native species where *Pteridium aquilinum* comprised about 6%, and about 2% exotic cover, which consisted of *G. aparine* and *B. tectorum* (Table 4).

A total of 114 herbaceous species, 96 native forbs, 11 native grasses, three exotic forbs, and four exotic grass species (Table 4) were found at the six sites. Species richness did not correlate closely with the N deposition gradient. The total number of species varied from 24 to 30 in four of the sites, with Dogwood, an intermediate pollution site, having 43 and Barton Flats having 57 herbaceous species in the 3 ha sampled at each site (Fig. 5B). The number of exotic species varied from one to three per site, so although the exotics made a major contribution to cover at Camp Paivika and Breezy Point, they did not

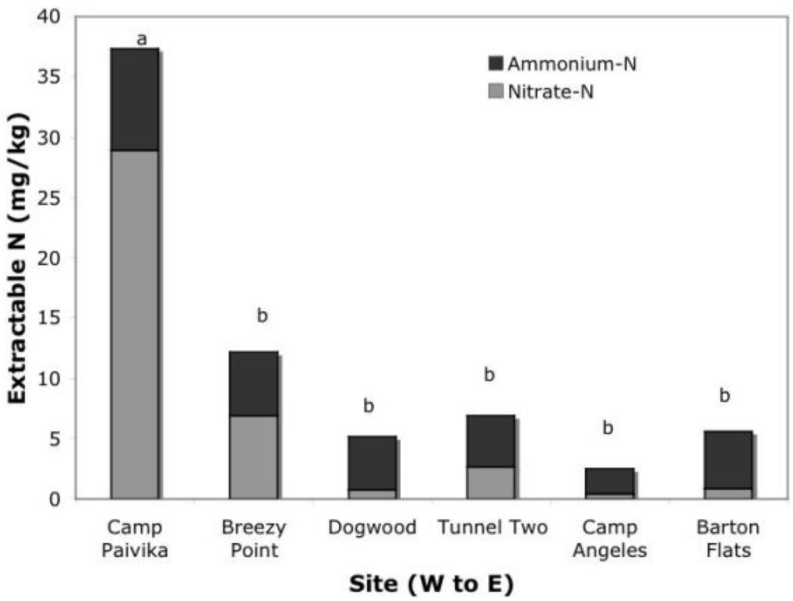

**A**

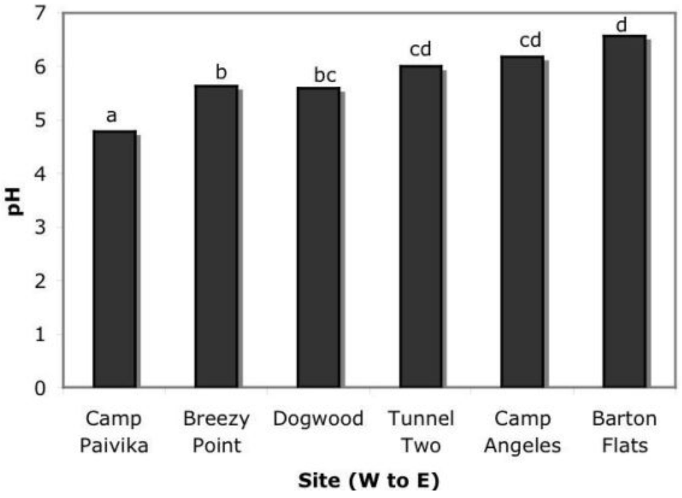

**B**

**FIGURE 2**. Extractable $NH_4^+$-N and $NO_3^-$-N as mg N/kg soil (A) and soil pH (B) in six sites along an air pollution gradient arranged from high to low pollution (W to E).

contribute much to richness. The most abundant native species at any one site was *P. aquilinum*, which occurred at three sites (Table 4). The annual *Claytonia perfoliata* had approximately 5% cover at Camp Paivika and lower at four other sites. *Nemophila menziesii* was next in abundance with more than 2% cover at Camp Paivika and Breezy Point, but it did not occur at any other site. *Elymus glaucus, Iris hartwegii,* and *Solidago californica* were the only species that occurred at all six sites, most often with considerably less than 1% cover. Nine of the native grass species occurred at Barton Flats, five at Dogwood, and one to four at the other sites. An analysis of similarity of the sites calculated using Sorenson's index (2w/a+b, where w = # species in common, a = species in sample a, b = species in sample b), showed that sites varied in percent species overlap from 25 to 71%. As expected, sites that were adjacent along the gradient had the highest species overlap, while those farther away had lower overlap.

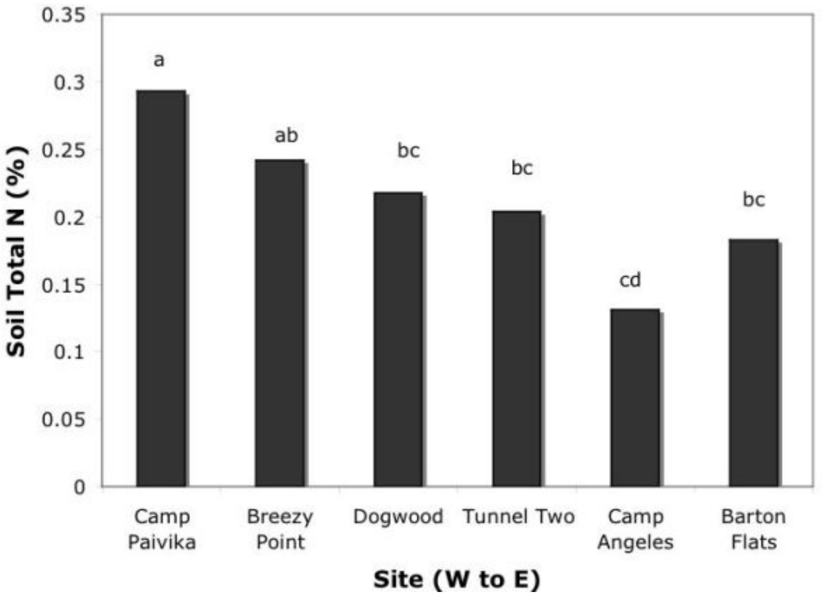

**A**

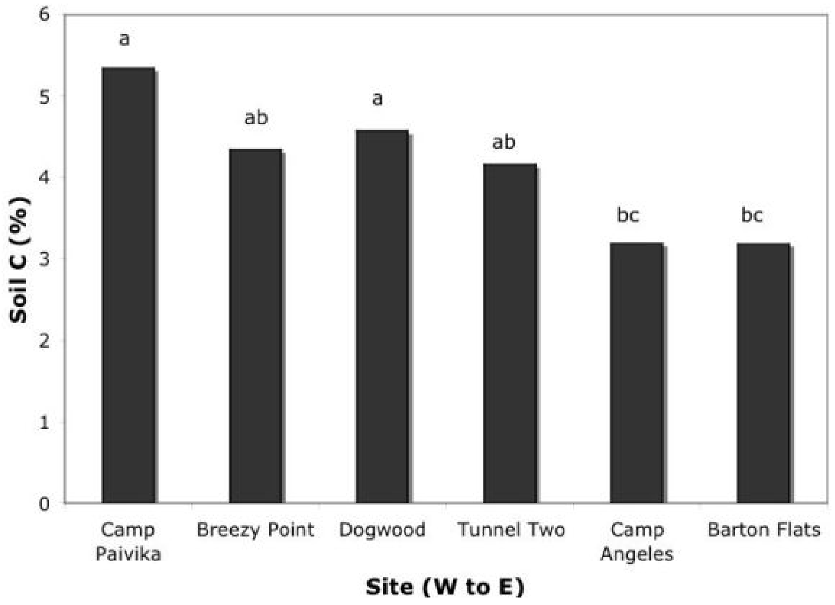

**B**

**FIGURE 3.** Percent total soil N (A) and C (B) in six sites along an air pollution gradient arranged from high to low pollution (W to E).

Comparisons of the species lists from 1973 and 2003 suggest that major changes have occurred in the understory vegetation of the mixed conifer forest over the past 30 years (Table 5). More species occurred in 1973 than in 2003 in three of six sites, including the two westernmost sites. Two sites had little change in total species number (Dogwood and Tunnel Two), and one, Barton Flats, had more species in 2003. There was considerable turnover of species even in sites that had the same number of species in 1973 and 2003, with species in common between both years ranging from 42–67% among the sites. A separate analysis was done to determine whether perennial vs. annual species persisted longer over the 30 years, but on average over the six sites, 41% of the annuals persisted and 43% of the perennials.

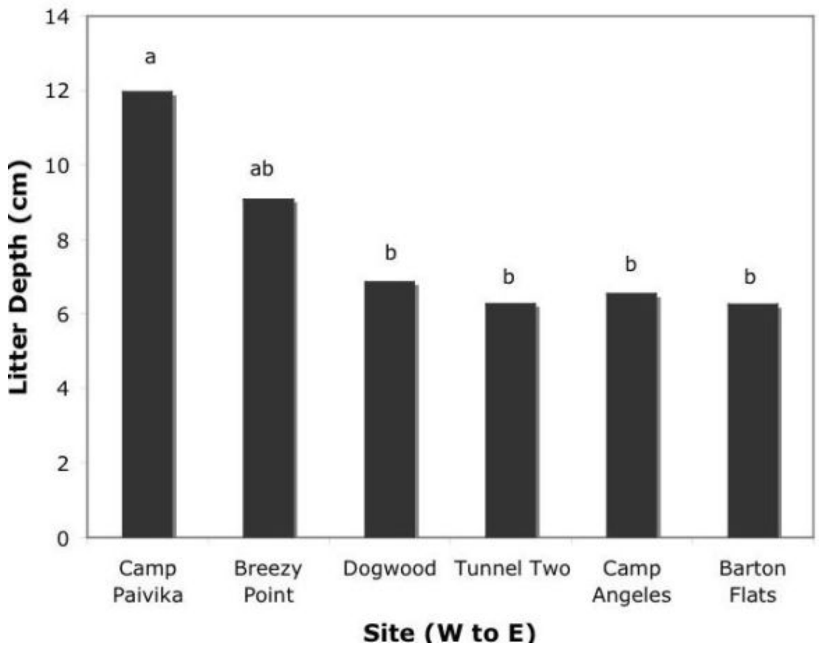

**FIGURE 4.** Depth of undecomposed soil surface litter (cm) in six sites along an air pollution gradient arranged from high to low pollution (W to E).

**TABLE 3**
**Tree Count per Hectare of Trees <10 cm dbh in 2003, >10 cm dbh in 2003 and 1973; and Basal Area in 2003 and 1973 at Six Sites Along an Air Pollution Gradient**

| | Number of Trees | | | | |
|---|---|---|---|---|---|
| | <10 cm dbh | >10 cm dbh | | Basal Area ($m^2 ha^{-1}$) | |
| Site | 2003 | 2003 | 1973* | 2003 | 1973* |
| Camp Paivika | nd** | 400 | nd** | 50.2 | 30.5 |
| Breezy Point | 685 | 517 | 443 | 52.7 | 44.1 |
| Dogwood | 1160 | 661 | 279 | 70.5 | 24.7 |
| Tunnel Two | 1019 | 435 | 185 | 35.5 | 23.9 |
| Camp Angeles | 835 | 323 | 210 | 43.5 | 50.6 |
| Barton Flats | 421 | 528 | 358 | 47.2 | 33.6 |

* From Miller et al.[24].

** nd = no data.

## DISCUSSION

### Soil and Air Pollution

Soil C and N data were collected in five of the six sites (with the exception of Camp Angeles) in the mid-1970s[27]. The values averaged over the five sites were total C, 2.21% (S.E. = 0.9) and total N, 0.089% (0.037). These data showed no trend along the air pollution gradient at that time as do present-day soil measurements, and they are considerably lower than the 2006 values. The lower values are most likely

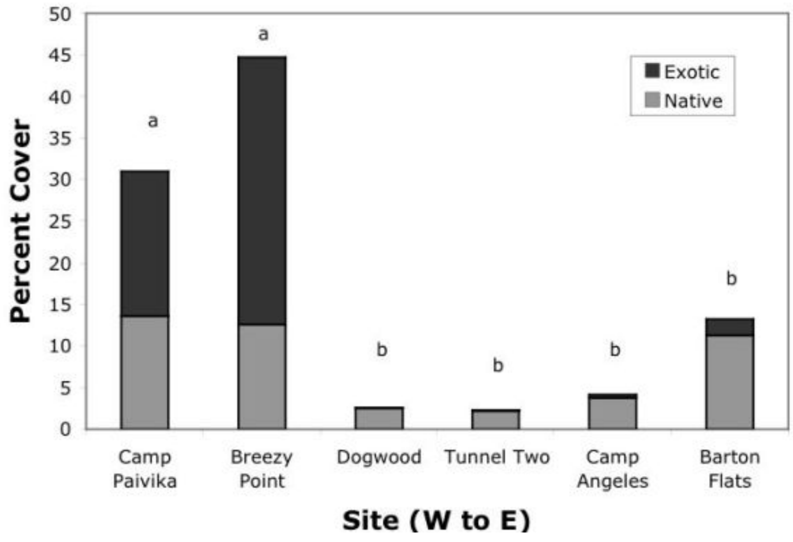

A

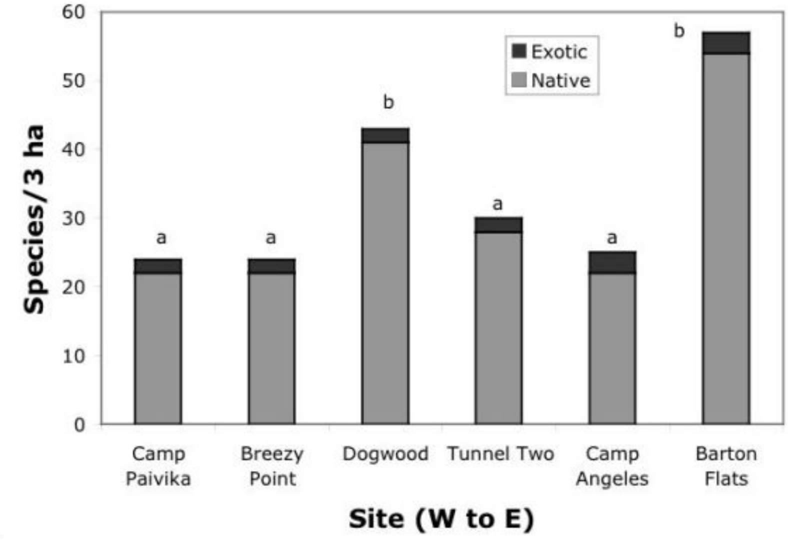

B

**FIGURE 5**. Percent cover (A) and richness (number of species per 3 ha) (B) of understory herbs in six sites along an air pollution gradient arranged from high to low pollution (W to E).

because of different coring depths. We cored to 5 cm to detect deposited N that accumulates on surface soils during the dry season[11]. Miller et al. most likely cored to the standard 15-cm depth (although the methods are not given[28]), so concentrated surface soil N and C would have been diluted by deeper, lower concentrations. However, there was no gradient in either total N or C in the 1977 report, suggesting that the major impacts of air pollution had not yet affected those soil characteristics. Grulke et al. also reported a low pH at Camp Paivika of 3.98, while a site close to Dogwood (Strawberry Peak) had a pH of 5.77[29], supporting the present-day gradient. Extractable N values from the 1990s also corroborate the existence of a soil pollution gradient that was impacted by N deposition, and high litter fall and soil C were also reported from Camp Paivika[8,13].

**TABLE 4**
**Percent Cover of Understory Species in Six Sites Along an Air Pollution Gradient in 2003, and 1973 Occurrence[24]**

| | | Camp Paivika | | Breezy Point | | Dogwood | | Tunnel Two | | Camp Angeles | | Barton Flats | |
|---|---|---|---|---|---|---|---|---|---|---|---|---|---|
| **Species** | **A/P** | **2003** | **1973** | **2003** | **1973** | **2003** | **1973** | **2003** | **1973** | **2003** | **1973** | **2003** | **1973** |
| **Exotic Forbs** | | | | | | | | | | | | | |
| *Galium aparine* | A | 16.3 | x | 32.1 | x | | | p | | | | 1.46 | |
| *Sisymbrium altissiumum* | A | | | | | | | | | | | | x |
| *Verbascum thapsis* | P | | | | | | | | | | | | x |
| **Exotic Grasses** | | | | | | | | | | | | | |
| *Bromus diandrus* | A | 1.07 | | 0.02 | x | | | | | 0.21 | | | |
| *B. tectorum* | A | | x | | x | 0.001 | x | 0.03 | x | 0.20 | x | 0.47 | x |
| *Elytrigia intermedium* | P | | | | | | | | | | x | | |
| *Poa pratensis* | P | | | | | | | | | | | 0.01 | |
| **Native Forbs/Subshrubs** | | | | | | | | | | | | | |
| *Achillea lanulosa* | P | | x | | | | | | | | | 0.25 | x |
| *Agoseris retrorsa* | P | | x | | x | p | | 0.36 | x | | | 0.00 | x |
| *Antennaria dimorpha* | P | | | | | | | | x | | | | |
| *A. rosea* | P | | | | | | | | | | | p | |
| *Arabis pulchra* | P | | | | | 0.01 | | | | | | 0.001 | |
| *A. rectissima* | P | | | | | p | x | | | | | | |
| *Artemisia douglasiana* | P | | | 3.81 | x | | | | | | | | |
| *A. dracunculus* | P | | | | x | | | | | | | 0.01 | x |
| *A. ludoviciana* | P | | | | | | | | | | | 0.15 | x |
| *Asclepias eriocarpa* | P | | | | x | | | | | 0.09 | x | | |
| *Bloomeria crocea* | P | | x | 0.001 | x | 0.02 | x | 0.03 | x | | | 0.01 | |
| *Calochortus invenustus* | P | | | | | p | | | | p | | 0.001 | |
| *Calystegia occidentalis* | P | 0.01 | x | 0.19 | | | x | | | | x | 0.06 | x |
| *Camissonia spp.* | P | | | | | | | | | | | 0.01 | |
| *Carex multicaulis* | P | 0.04 | x | | | 0.01 | x | 0.11 | x | 0.04 | x | 0.23 | x |
| *C. rossii* | P | | | | | | | | | | x | | |
| *Caulanthus amplexicaulis* | A | | | | | 0.20 | x | | | | | | |
| *Chaenactis santolinoides* | P | | | | | | | | x | | | | |
| *Chenopodium fremontii* | A | | | | | | | | | | | p | |
| *Chimaphila umbellata* | P | | | | | | | | | 0.01 | | | |
| *Cirsium occidentale ssp. californicum* | P | | | | | | | | | | | | x |
| *Clarkia rhomboidea* | A | 0.04 | x | 0.01 | x | 0.03 | | 0.002 | | | | | |
| *Claytonia lanceolata* | P | | | | | | | | | | | 0.23 | |
| *C. perfoliata* | A | 5.61 | x | 1.93 | | 0.71 | x | 0.001 | | | | 0.19 | |
| *Collinsia childii* | A | 0.38 | x | 1.16 | x | 0.00 | | p | | | | | |
| *C. heteorphylla* | A | | | 0.02 | | | | | | | | | |
| *Collomia grandiflora* | A | 0.00 | x | 0.08 | x | | | | | | | | |
| *Corallorhiza maculata* | P | | | | | | | | | | | p | |
| *Cordylanthus nevlnii* | A | | | | | 0.001 | | p | | | x | | |
| *Cryptantha affinis* | A | | x | | x | | | p | | p | | | |
| *Cryptantha simulans* | A | | | | | 0.03 | x | 0.16 | | 0.03 | x | 0.03 | |
| *Delphinium parryi* | P | | | p | | | | | | | | 0.002 | |
| *Dichelostemma capitatum* | P | 0.01 | x | 0.15 | x | 0.01 | x | p | | p | | 0.08 | x |
| *Epilobium canum* | P | | | | x | | | | x | 0.001 | x | | |
| *E. ciliatum* | A | | | | | | | | | | | 0.01 | |
| *Equisetum hyemale* | P | | | | | | | | | | | p | |

### TABLE 4 (continued)

| Species | A/P | Camp Paivika | | Breezy Point | | Dogwood | | Tunnel Two | | Camp Angeles | | Barton Flats | |
|---|---|---|---|---|---|---|---|---|---|---|---|---|---|
| | | 2003 | 1973 | 2003 | 1973 | 2003 | 1973 | 2003 | 1973 | 2003 | 1973 | 2003 | 1973 |
| *Eriastrum densiflorum* | A | | | | | | | | | | | 0.001 | x |
| *Erigeron foliosus* | P | | x | 0.08 | x | 0.001 | x | 0.001 | x | | x | 0.06 | x |
| *Eriogonum molestum* | A | | | | | p | x | | | | | | |
| *E. nudum* | P | | | | | 0.31 | | | | | | 0.01 | |
| *E. saxatile* | P | | | | | | x | | | | | | |
| *E. umbellatum* | p | | | | | | | | | | x | | |
| *E. wrightii* | P | | | | | | | | | | | | x |
| *Eriophyllum confertiflorum* | P | | | | | | | | x | | | | |
| *E. lanatum ssp. obovatum* | P | | | | | | x | | | | | | |
| *Erysimum capitatum* | P | 0.01 | x | p | x | p | x | p | | 1.20 | x | 0.01 | x |
| *Euphorbia palmeri* | A | | | | | | | | | | | | x |
| *Fragaria vesca* | P | | | 0.40 | | | | | | | | p | |
| *Galium angustifolium* | P | | | | | | | | | | | 0.07 | x |
| *G. porrigens* | P | 1.33 | x | | | | | | | | | | |
| *Galium spp.* | P | | | | | | | | x | | | | x |
| *G. trifidum* | P | | | 0.47 | x | | | | | | | | |
| *Gayophytum diffusum* | A | | x | | x | | x | 0.00 | x | 0.20 | x | 0.02 | x |
| *Gilia capitita* | A | | x | | x | | | | | | | | |
| *G. splendens* | A | | | | | 0.62 | x | | | | | | |
| *Gnaphalium stramineum* | P | | | | | | x | | x | | x | | x |
| *Hieracium albiflorum* | P | | | | | | x | 0.07 | x | | | 0.03 | |
| *Horkelia rydbergii* | P | | | | | 0.31 | x | | | | | p | |
| *Iris hartwegii* | P | 0.07 | x | p | x | 0.13 | x | 0.17 | x | 0.09 | x | 0.26 | x |
| *Lathyrus vestitus* | P | | x | | x | | | | | | | | |
| *Lessingia filaginifolia* | P | | | | x | p | x | | | 0.31 | x | p | x |
| *Linanthus breviculis* | A | | | | | | x | | x | | | | |
| *L. ciliatus* | A | | | | | 0.02 | x | 0.06 | | | | | |
| *L. nuttalii* | P | | | | | | | | | | | 0.66 | x |
| *Lithophragma affine* | P | 0.03 | | 0.01 | | | | | | | | | |
| *Lotus argophyllus* | P | | | | | p | x | 0.001 | x | 0.03 | x | 0.02 | |
| *L. crassifolius* | P | | | | | | | | | 0.10 | x | | |
| *L. grandiflorus* | P | | | | x | | | | | | x | | |
| *L. nevadensis* | P | | | | | p | | | | | | p | |
| *L. purshianus* | A | | | | | | | | x | | | | x |
| *Lupinus andersonii* | P | | | | x | | x | | | | | | |
| *L. excubitus* | P | | | | | p | x | p | X | | | | |
| *Madia elegans* | A | | | | x | p | | | | | x | | |
| *Mentzelia montana* | P | | | | | | | | | | | 0.01 | |
| *Monardella lanceolata* | P | | | | | 0.01 | x | | | | | 0.001 | x |
| *Nemophila menziesii* | A | 2.56 | x | 2.65 | x | | | | | | | | |
| *Osmorhiza chilense* | P | 0.57 | x | 0.82 | | 0.02 | x | p | | | | 0.004 | |
| *Pedicularis densiflora* | P | | | | | | | 0.001 | x | | x | | |
| *Penstemon grinnellii* | P | | | | | | | | | 0.01 | x | | |
| *P. labrosus* | P | | | | | p | x | | x | 0.22 | | | x |
| *Phacelia curvipes* | A | p | | | | 0.07 | x | | x | p | | 0.04 | |
| *Piperia elegans* | P | | | | x | p | | 0.05 | | p | | | |
| *Polygonum douglasii* | A | | | | | | | | | | | | x |
| *Polystichum munitum* | P | | | | | | | | | | | p | |
| *Potentilla glandulosa* | P | p | x | | | | | | | | | 0.29 | |
| *Pteridium aquilinum* | P | 0.31 | x | 0.07 | x | | x | | | | x | 6.29 | x |

### TABLE 4 (continued)

| Species | A/P | Camp Paivika | | Breezy Point | | Dogwood | | Tunnel Two | | Camp Angeles | | Barton Flats | |
|---|---|---|---|---|---|---|---|---|---|---|---|---|---|
| | | 2003 | 1973 | 2003 | 1973 | 2003 | 1973 | 2003 | 1973 | 2003 | 1973 | 2003 | 1973 |
| *Pterospora andromedia* | P | | x | | | | | | | | | | |
| *Pyrola picta* | P | | | | | | x | | | | | | |
| *Scutellaria siphocampyloides* | P | | | | x | | x | | | | | 0.02 | x |
| *Silene verecunda* | P | | | | | 0.001 | x | | x | | | 0.01 | |
| *Solanum xanti* | P | 0.35 | x | | | | | | | | | | |
| *Solidago californicum* | P | 0.07 | x | 0.53 | x | p | x | 0.08 | | 0.13 | x | 0.19 | x |
| *Stephanomeria virgata* | A | | | | | 0.001 | x | | | | x | | |
| *Trichostema micrantum* | P | | | | | p | | | | | | | |
| *Vicia americana* | P | 0.60 | x | 0.17 | x | 0.02 | x | 0.27 | x | | | 0.39 | x |
| *Viola pinetorum* | P | | | | | p | | | | | | | |
| *V. purpurea* | P | 0.01 | | | | | | p | | | | | |
| **Native Grasses** | | | | | | | | | | | | | |
| *Achnatherum occidentale* | P | | | | | | x | | | | x | | |
| *Bromus carinatus* | P | 0.24 | x | | x | 0.13 | x | 0.63 | x | 0.01 | x | 0.29 | x |
| *B. orcuttianus* | P | | | | x | | | | x | | x | p | |
| *Elymus elymoides* | P | | | | | 0.001 | x | | x | p | x | 0.001 | x |
| *E. glaucus* | P | 1.36 | | 0.02 | x | 0.05 | x | 0.002 | x | 0.94 | x | 1.28 | x |
| *Festuca rubra* | P | | | | | | | | | | | 0.02 | x |
| *Festuca spp.* | P | | | | x | 0.05 | | 0.07 | | | | 0.001 | |
| *Juncus balticus* | P | | | | | | | | | | | 0.01 | |
| *Koeleria macrantha* | P | | | | | | x | | | | x | 0.09 | x |
| *Melica aristata* | P | | | | x | | x | | | | x | | |
| *Poa fendleriana* | P | | | | | p | | 0.27 | | | | 0.03 | x |

*Note:* A = annual; P = perennial; p = present in 1-ha plots, but not in sample quadrats in 2003; x = present in 1973. Nomenclature from Hickman (1993)[39].

### TABLE 5
## Changes in Understory Species Richness, 1973–2003, at Six Sites Across an Atmospheric Pollution Gradient, Highest Rates of Pollution to Lowest, in the San Bernardino Mountains

| Site | % of Plant Species | | | No. of Species | | |
|---|---|---|---|---|---|---|
| | % in Common | % Present Only in 2003 | % Present Only in 1973 | Total No. in 2003 | Total No. in 1973 | % Change |
| Camp Paivika | 67 | 18 | 33 | 22 | 27 | –19 |
| Breezy Point | 42 | 32 | 47 | 22 | 36 | –39 |
| Dogwood | 67 | 34 | 33 | 41 | 42 | –2 |
| Tunnel Two | 46 | 54 | 54 | 28 | 26 | 8 |
| Camp Angeles | 50 | 32 | 50 | 22 | 30 | –27 |
| Barton Flats | 46 | 54 | 24 | 54 | 34 | 59 |

The reduction in ozone concentrations between 1973–1977 and 2002–2005 show the effectiveness of air pollution control regulations[5], and the ozone gradient is also relatively flattened compared to the earlier gradient. In the 1970s, the westernmost polluted site had 36% higher ozone concentration than sites to the east, while in the 2000s it was 14% higher. By contrast, there is a relatively steep gradient of atmospheric reactive N, 53% higher when $HNO_3$ and $NH_3$ are combined. This difference in patterns of ozone and N pollution may help to explain the patterns of impact on understory plants.

### Vegetation Response

The forest responded in the last 30 years with increased density and basal area of trees >10 cm dbh, and high densities of saplings and trees <10 cm dbh. As has been observed throughout the southern California mixed coniferous forests, fire suppression has led to stand densification[30]. An increase in the canopy over time may explain understory patterns. Dogwood, Tunnel Two, and Camp Angeles are shady sites that have experienced considerable stand densification, especially in the <10-cm size category, and currently have sparse understories. Camp Paivika and Breezy Point are also shady, but with less stand densification in the smaller size class (quantified only at Breezy Point). In addition to being at the mesic end of the gradient, they also receive more fog drip than other sites[9], which may be beneficial to herbaceous production especially when the winter rains cease and dewfall is the only precipitation input. The surprisingly high diversity at Barton Flats may be related to the drier climate. While the stand density is not lower than some of the other sites, it has a sparser canopy and more sunlight filtering to the forest floor. No light measurements were made to quantify this, but a drier climate may be a cause of a sparser canopy, even when the basal area is relatively high.

Another factor that may affect herbaceous species richness is the deep litter layer at Camp Paivika and to a lesser extent at Breezy Point. The litter layer consists primarily of pine needles that have dropped prematurely from trees that are still affected by ozone, even with the greatly reduced ozone over the last 30 years[31]. These are also the two sites that had high cover of the exotic annual *Galium aparine*, which apparently is not inhibited by the deep litter. Prior to the record drought of 2002, both of these sites also had an extensive cover of the native fern *Pteridium aquilinum* [Temple, unpublished observations], which still had litter lying on the surface of the pine needle litter at the time of sampling in 2003. During the 2006 visit to these two sites to collect soil samples, we noted that *P. aquilinum* was beginning to recover, although in small scattered patches. It is known worldwide as an invasive species that responds to various disturbances including fertilization, but it is native to the region. Fronds of *P. aquilinum* collected at Camp Paivika had significantly higher concentrations of N and lower C:N ratios than fronds collected at Barton Flats, indicating the greater availability of N at this site[12]. However, at Barton Flats, it was the most abundant understory species, indicating its broad ecological amplitude.

While ozone may have had an indirect impact on the herbaceous species by increasing tree litterfall, a direct impact is more difficult to detect. Among plants found in our samples, ozone injury has been observed in two perennials, *Artemisia douglasiana* and *A. dracunculus*, and two annuals, *Gayophytum diffusum* and *Cordylanthus rigidus*[3]. No ozone injury was detected in other annuals, including *Clarkia rhomboidea* and *Collomia grandiflora*. The canopy may benefit the herb layer by absorbing or interacting with some of the ozone and reducing the ozone exposure[32]. However, species have differential sensitivity to ozone, and some of the abundant species in the westernmost sites may be more resistant. Development of resistant populations of some weedy plant species in response to exposure to ozone has been reported[1,33]. However, such a phenomenon has not been observed at these highly polluted sites in the San Bernardino Mountains[3]. Ozone sensitivity studies on species such as *G. aparine* and *P. aquilinum* might help to explain their abundance.

The major mechanism by which elevated soil N affects plant community composition is to promote differential growth of species[34,35,36]. *Galium aparine* is known as a nitrophilous agricultural weed in Europe where it originates[37] and was very abundant in the two most polluted sites. Many grass species respond preferentially to N fertilization compared to forbs and shrubs, explaining why N deposition has resulted in grass dominance in heathlands in the Netherlands[15] and in coastal sage scrub in southern California[19,38]. Barton Flats was the site with the greatest grass richness, but also the lowest N pollution, and there was no increase in grass species richness between 1973 and 2003. The other sites had low cover of all grass species as would be expected of shady forest sites, including the invasive *Bromus* species that are highly responsive to N in lower-elevation sites[19,35]. *Nemophila menziesii* was the most abundant native forb at Camp Paivika and Breezy Point, and is also abundant in sites subject to high N deposition at lower elevations[19 and Allen unpublished].

There are several difficulties with comparing changes in species richness among the sites and between years. The original plot sizes assessed for native species in the 1973 studies were not published and, according to the report[22], were variable in size according to tree density. Thus a direct comparison of species richness between 1973 and 2003 is not technically correct, and can only be used as a guideline. Our best estimate of the 1973 methods used suggests that a smaller area was sampled in all of the sites than the 3 ha we sampled. We found more species at only one site, Barton Flats, and either fewer or an equal number at the others. We would have expected to find more species in all of the sites in 3 ha, so it is possible that five of six sites have declined in richness since 1973.

The second difficulty in comparing species richness responses to pollutants is that the sites are so very different in understory species composition, even though the two dominant tree species (*Pinus ponderosa* and *Quercus kelloggii*) were the same across the sites. Low similarity of 25–71% of distant vs. adjacent sites indicates both high alpha (or within-site diversity) and beta (or between-site diversity) across the landscape. In fact, the San Bernardino Mountains have only about 2% of the land area of California, but 25% of California plant species occur there [A. Sanders, personal communication, cited in 3]. The high richness also means that taking a statistically adequate sample becomes difficult, even though we sampled 3 ha per site. The species-area curve continues to increase as one moves across the gradient sampled here. In response to the problem of high richness at other sites, ecologists have recommended using functional guilds or grouping life-forms for analysis[36], as we have done by comparing exotic and native grasses and forbs, as well as comparing annual and perennial plant responses. With a large number of species in the available pool, the best determinant of response was change in species richness.

### CONCLUSIONS

Atmospheric N pollution and ozone were not linearly related to herb cover or richness of the six sites observed, as would be expected in a multidimensional gradient. However, there was clearly a detrimental effect of air pollution at the two westernmost sites, Camp Paivika and Breezy Point. Symptoms of ozone damage are still severe on pine trees, but were observed on only a few herb species[3]. A negative ozone effect on the understory may have been indirect through the increased pine litterfall. Deep litter may prohibit establishment of native herbs, while the exotic annual *Galium aparine* was abundant even in deep litter. This species has an indeterminate growth form that may enable it to establish in deep litter. Coupled with the increased litter depth were decreased pH, increased soil N, and higher precipitation that are also suitable for growth of *G. aparine*[37]. Furthermore, Camp Paivika and Breezy Point both lost species since 1973. The reason for fewer species in 1973 at Camp Angeles is unclear, although this site is near suburban development. These sites are continuously undergoing environmental changes. The drought of 2002 caused tree mortality to some extent at all sites through 2004. This opened the canopy to invasive annual grasses. A portion of the Camp Paivika site was burned in October 2003 (after the summer 2003 sample period), and formerly shady sites are now covered by exotic annual brome grasses. Clearing of dead trees and brush in 2005 to reduce the fuel load is further exposing the soil to invasive species, and road and suburban developments are encroaching the Tunnel Two and Camp Angeles sites. The highly diverse herbaceous species clearly are threatened by air pollution and other disturbances, and are worthy of protection in forest conservation plans.

### ACKNOWLEDGMENTS

This research was funded by USEPA Grant #R-82890101-0 and NSF-DEB 04-21530. We are grateful to Hally Anderson, Ian Gillespie, Sheila Kee, Brooke Lyons, Mitchell Provence, Greg Smith, and Tracy Tennant for field assistance, and Andrew Sanders, Curator of the UCR Herbarium, for species identification.

## References

1. Davison, A.W. and Barnes, J.D. (1998) Effects of ozone on wild plants. *New Phytol*. **139,** 135–151.
2. Gilliam, F.S. (2006) Response of the herbaceous layer of forest ecosystems to excess nitrogen deposition. *J. Ecol*. **94**, 1176–1191.
3. Temple, P.J., Miller, P.R., and McBride, J.R. (1999) Effects of ozone on understory vegetation in the mixed conifer forest. In *Oxidant Air Pollution Impacts in the Montane Forests of Southern California*. Springer, New York. pp. 208–222.
4. Miller, P.R. (1973) Oxidant induced community changes in a mixed conifer forest. In Air Pollution Damage to Vegetation. *Adv. Chem. Ser.* **122**, 101–117.
5. Miller, P.R. and McBride, J.R., Eds. (1999) *Oxidant Air Pollution Impacts in the Montane Forests of Southern California*. Springer, New York.
6. Fenn, M.E., Haeuber, R., Tonnesen, G.S., Baron, J.S., Grossman-Clarke, S., Hope, D., Jaffe, D.A., Copeland, S., Geiser, L., and Rueth, H.M. (2003) Nitrogen emissions, deposition, and monitoring in the western United States. *BioScience* **53,** 391–403.
7. Bytnerowicz, A., Miller, P.R., and Olszyk, D.M. (1987) Dry deposition of nitrate, ammonium and sulfate to a Ceanothus-Crassifolius canopy and surrogate surfaces. *Atmos. Environ.* **21**, 1749–1757.
8. Fenn, M.E., Poth, M.A., and Johnson, D.W. (1996) Evidence for nitrogen saturation in the San Bernardino Mountains in southern California. *For. Ecol. Manage*. **82,** 211–230.
9. Fenn, M.E., Poth, M.A., Schilling, S.L., and Grainger, D.B. (2000) Throughfall and fog deposition of nitrogen and sulfur at an N-limited and N-saturated site in the San Bernardino Mountains, southern California. *Can. J. For. Res.* **30,** 1476–1488.
10. Fenn, M.E., Baron, J.S., Allen, E.B., Rueth, H.M., Nydick, K.R., Geiser, L., Bowman, W.D., Sickman, J.O., Meixner, T., Johnson, D.W., and Neitlich, P. (2003) Ecological effects of nitrogen deposition in the western United States. *BioScience* **53**, 404–420.
11. Padgett, P.E., Allen, E.B., Bytnerowicz, A., and Minich, R.A. (1999) Changes in soil inorganic nitrogen as related to atmospheric nitrogenous pollutants in southern California. *Atmos. Environ.* **33**, 769–781.
12. Fenn, M.E., Poth, M.A., Bytnerowicz, A., Sickman, J.O., Takemoto, B.K., Bytnerowicz, A., Arbaugh, M.J., and Alonzo, R. (2003) Effects of ozone, nitrogen deposition, and other stressors on montane ecosystems in the Sierra Nevada. In *Ozone Air Pollution in the Sierra Nevada: Distribution and Effects on Forests.* Elsevier Science, Oxford. pp. 111–155.
13. Miller, P.R., Rechel, J., Miller, P.R., and McBride, J.R. (1999) Temporal changes in crown condition indices, needle litterfall, and collateral needle injuries of ponderosa and Jeffrey pines. In *Oxidant Air Pollution Impacts in the Montane Forests of Southern California*. Springer, New York. pp. 164–178.
14. Fenn, M.E. and Poth, M.A. (2001) A case study of nitrogen saturation in western U.S. forests. *TheScientificWorldJOURNAL* **1**, 433–439.
15. Bobbink, R., Hornung, M., and Roelofs, J.G.M. (1998) The effects of air-borne nitrogen pollutants on species diversity in natural and semi-natural European vegetation. *J. Ecol.* **86,** 717–738.
16. Stevens, C.J., Dise, N.B., Mountford, J.O., and Gowing, D.J. (2004) Impact of nitrogen deposition on the species richness of grasslands. *Science* **303,** 1876–1879.
17. Brooks, M.L. (2003) Effects of increased soil nitrogen on the dominance of alien annual plants in the Mojave Desert. *J. Appl. Ecol.* **40,** 344–353.
18. Allen, E.B., Rao, L.E., Steers, R.J., Bytnerowicz, A., Fenn, M.E., Webb, R.H., Fenstermaker, L.F., Heaton, J.S., Hughson, D.L., McDonald, E.V., and Miller, D.M. (2007) Impacts of atmospheric nitrogen deposition on vegetation and soils in Joshua Tree National Park. In *The Mojave Desert: Ecosystem Processes and Sustainability*. University of Nevada Press, Las Vegas, in press.
19. Allen, E.B. (2002) Restoration of Artemisia Shrublands Invaded by Exotic Annual Bromus: A Comparison between Southern California and the Intermountain Region. Hild, A.L., Shaw, N.L., Meyer, S.E., Schupp, E.W., and Booth, T., Eds. Rocky Mountain Research Station, Forest Service, U.S. Department of Agriculture. pp. 9–17.
20. Allen, E.B., Padgett, P.E., Bytnerowicz, A., and Minnich, R.A. (1996) Nitrogen Deposition Effects on Coastal Sage Vegetation of Southern California. USDA Forest Service, Pacific Southwest Research Station, Riverside, CA. pp. 131–140.

21. D'Antonio, C.M. and Vitousek, P.M. (1992) Biological invasions by exotic grasses, the grass fire cycle, and global change. *Annu. Rev. Ecol. Syst*. **23,** 63–87.
22. McBride, J.R. and Semion, V. (1976) Vegetation Sub-Committee Progress Report, June 1975–June 1976. Department of Forestry and Conservation, University of California, Berkeley.
23. Fenn, M.E. and Poth, M.A. (1999) Temporal and spatial trends in streamwater nitrate concentrations in the San Bernardino Mountains, southern California. *J. Environ. Qual.* **28,** 822–836.
24. Miller, P.R., McBride, J.R., Schilling, S.L., Gomez, A.P., Olson, R.K., and Lefohn, A.S. (1989) Trends of ozone damage to conifer forests between 1974 and 1988 in the San Bernardino Mountains of southern California. In *Effects of Air Pollution on Western Forests*. Air Waste Management Association, Pittsburgh, PA. TR-16. pp. 309–323.
25. Bytnerowicz, A. (2007) Air pollution distribution patterns in the San Bernardino Mountains of southern California: a 40-year perspective. *TheScientificWorldJOURNAL* **7(S1)**, 98-109. DOI: 10.1100/tsw.2007.57.
26. Taylor, O.C. (1974) Oxidant Air Pollution Effects on a Western Coniferous Forest Ecosystem, Task D. Statewide Air Pollution Research Center, University of California, Riverside. Unpublished Report.
27. Poth, M.A., Wohlgemuth, P., Miller, P.R., and McBride, J.R. (1999) Geography, geology, geomorphology, and forest soils. In *Oxidant Air Pollution Impacts in the Montane Forests of Southern California*. Springer, New York. pp. 7–27.
28. Miller, P.R., Kickert, R.N., Taylor, O.C., Arkely, R.J., Cobb, F.W., Jr., Dahlsten, D.L., Gersper, P.J., Luck, J.R., McBride, J.R., Parmeter, J.R., Jr., Wenz, J.M., White, M., and Wilcox, W.W. (1977) Photochecmical Oxidant Air Pollutant Effects on a Mixed Conifer Forest Ecosystem-A Progress Report, 1975–1976. U.S. Environmental Protection Agency, Corvallis, OR.
29. Grulke, N.E., Andersen, C.P., Fenn, M.E., and Miller, P.R. (1998) Ozone exposure and nitrogen deposition lowers root biomass of ponderosa pine in the San Bernardino Mountains, California. *Environ. Pollut.* **103,** 63–73.
30. Minnich, R.A., Barbour, M.G., Burk, J.H., and Fernau, R.F. (1995) 60 years of change in Californian conifer forests of the San-Bernardino Mountains. *Conserv. Biol*. **9,** 902–914.
31. Grulke, N.E. and Balduman, L. (1999) Deciduous conifers: high N deposition and O-3 exposure effects on growth and biomass allocation in ponderosa pine. *Water Air Soil Pollut.* **116,** 235–248.
32. Bytnerowicz, A., Fenn, M.E., Miller, P.R., Arbaugh, M.J., Miller, P.R., and McBride, J.R. (1999) Wet and dry pollutant deposition to the mixed conifer forest. In *Oxidant Air Pollution Impacts in the Montane Forests of Southern California.* Springer, New York. pp. 235–269.
33. Davison, A.W. and Reiling, K. (1995) A rapid change in ozone resistance of Plantago major after summers with high ozone concentrations. *New Phytol*. **131,** 337–344.
34. Padgett, P.E. and Allen, E.B. (1999) Differential responses to nitrogen fertilization in native shrubs and exotic annuals common to Mediterranean coastal sage scrub of California. *Plant Ecol.* **144**, 93–101.
35. Yoshida, L.C. and Allen, E.B. (2004) N-15 uptake by mycorrhizal native and invasive plants from a N-eutrophied shrubland: a greenhouse experiment. *Biol. Fertil. Soils* **39**, 243–248.
36. Suding, K.N., Collins, S.L., Gough, L., Clark, C., Cleland, E.E., Gross, K.L., Milchunas, D.G., and Pennings, S. (2005) Functional- and abundance-based mechanisms explain diversity loss due to N fertilization. *Proc. Natl. Acad. Sci. U. S. A.* **102,** 4387–4392.
37. Dunker, M. and Nordmeyer, H. (2000) Reasons for the distribution of weed species on arable fields - field and greenhouse experiments concerning the influence of soil properties. *Z. Pflanzenkr. Pflanzenschutz* 55–62.
38. Allen, E.B., Sirulnik, A., Egerton-Warburton, L., Kee, S., Bytnerowicz, A., Padgett, P.E., Temple, P., Fenn, M.E., Poth, M.A., Meixner, T., Kus, B.E., and Beyers, J.L. (2005) Air pollution and vegetation change in California shrublands. In Planning for Biodiversity: Bringing Research and Management Together. Pacific Southwest Research Station, Forest Service, U.S. Department of Agriculture, Albany, CA. pp. 79–96.
39. Hickman, J.C., Ed. 1993. The Jepson manual. Higher plants of California. University of California Press, Berkeley.
